# An open image dataset of Indonesian soybean seed varieties (Anjasmoro, Grobogan, DEGA-1) for agricultural research and machine learning applications

**DOI:** 10.1016/j.dib.2026.112524

**Published:** 2026-02-03

**Authors:** Diana Sofia Hanafiah, Rahmatika Alfi, Anggria Lestami, Fanindia Purnamasari, Rossy Nurhasanah, Muhammad Ariyo Syahraza, Muhammad Azis Saputra, Usman Ismail Pane, Steven Manurung, Keisya, Yunus Tio Buntoro, Josua Peter Corda, Gali Rakasiwi

**Affiliations:** a Faculty of Agriculture, Universitas Sumatera Utara, Medan 20155, Indonesia; b Faculty of Computer Science and Information Technology, Universitas Sumatera Utara, Medan 20155, Indonesia

**Keywords:** Soybean, Plant breeding, Computer vision, Agricultural imaging, Segmentation

## Abstract

Soybean (*Glycine* max L.*)* performs an important position as a main resource of protein in Indonesia. Its quality and productivity can be assessed based on the characteristics of its seed. Accordingly, the identification process through the observation of soybean seed traits is a crucial step in plant breeding and quality assurance. Manual approaches rely on manual observation, which is subjective, prone to human error and time-consuming. With the improvement of artificial intelligence, automated seed identification has appeared as a potential solution. However, progress is constrained by the lack of open and standardized image datasets, especially for locally bred varieties in developing countries. To address this gap, we propose an open image dataset of Indonesian soybean seeds from three widely cultivated and plant-bred varieties: Anjasmoro, Grobogan, and DEGA-1. The dataset consists of high-resolution seed images captured with an Epson L360 flatbed scanner, with the optical resolution fixed at 800 dots per inch, yielding images of 6800 × 9359 pixels. All raw images are saved in JPG format. No manually segmentation masks are released in this version, instead of using Deeplab V3+ with MobileNet as backbone to enable the automated seed image segmentation. The curated dataset is intended to support a broad range of applications, including computer vision tasks such as image classification and segmentation, as well as research in plant breeding, seed quality assessment, and agricultural informatics. By providing a standardized and publicly accessible resource, this dataset contributes to the advancement of interdisciplinary studies at the intersection of agriculture and artificial intelligence.

Specifications Table

**Table:** 

Subject	Plant Breeding, Computer Vision, Agricultural Imaging
Specific subject area	Computer vision for seed phenotyping and varieties classification using scanner-based imagery.
Type of data	The dataset consists of digital images of soybean seeds from three Indonesian plant-bred varieties (Anjasmoro, Grobogan, and DEGA-1). All images are provided in JPG format as RGB color images.
Data collection	Seed images were captured by using high-resolution scanner of Epson L360 at 800 dpi, resulting in image dimensions of 6800 × 9359 pixel under standardized condition. Seeds placed flat on the glass with the total of 119 time scanning, yielding 12,683 seed instances collected
Data source location	The data were collected at Biotechnology Laboratory, Faculty of Agriculture, Universitas Sumatera Utara, Medan, Indonesia
Data accessibility	Repository name: Mendeley Data Data identification number: DOI: 10.17632/c733bjz4m3.3 Direct URL to data: https://data.mendeley.com/datasets/c733bjz4m3/3
Related research article	none.

## 1 Value of the Data

• The dataset introduces standardized, high-resolution seed imagery for three elite Indonesian soybean varieties, namely Anjasmoro, Grobogan, and DEGA-1, which have not previously been represented in global soybean image repositories. It differs from existing published datasets such as [[1], [2]], which mainly focus on defect classification and provide images of individual soybean seeds grouped into five commercial-quality categories, including intact, immature, skin-damaged, spotted, and broken. The present dataset is designed to capture varietal-level and phenotypic characteristics. By focusing on tropical soybean varieties, this dataset fills an important gap in global soybean image resources, since the morphological traits of tropical varieties, influenced by regional climate and environmental conditions, are still limited in existing datasets.
• The dataset comprises 119 high-resolution raw scanned images and 12.683 segmented images of individual seeds, structured into six folders (three per-variety raw scans and three segmentation folders). This directory organization enables straightforward filtering by variety, stratified sampling, and the construction of reproducible train/validation/test splits, which can address the broader need for standardized acquisition protocols in agricultural imaging and complementing existing soybean datasets.
• The dataset emphasizes phenotypic and varietal representation, documenting three elite Indonesian tropical varieties that remain absent from global datasets. Moreover, most existing public soybean datasets primarily feature varieties from the United States and China, resulting in limited representation of tropical phenotypes [3] Tropical soybean varieties exhibit distinctive morphological traits driven by differences in climate and environmental conditions.
• Beyond its role as a high-quality imaging corpus, the dataset is intentionally designed to support broad reuse across a wide range of computer-vision tasks, including variety classification, instance detection or segmentation (with user-provided annotations), seed counting, and morphology-aware feature extraction encompassing shape, texture, and color.
• The controlled scanner environment further renders the dataset a reliable source for transfer learning and domain adaptation, enabling systematic evaluation of model robustness when deployed in field, smartphone, or conveyor-belt imaging scenarios. Owing to its high resolution and clean visual structure, the dataset also facilitates data-efficient training strategies and supervised learning. With its well-defined scope and the availability of both raw scan images and individually segmented seeds, this dataset establishes a new and distinctive benchmark for digital agriculture research, the breeding of superior tropical varieties, and the development of machine-learning models with enhanced robustness to global phenotypic diversity.

## 2 Background

The development of automated seed classification systems has become increasingly important for agricultural quality control and breeding programs, particularly for economically significant crops like soybean. Traditional manual seed sorting methods are time-consuming, subjective, and prone to human error. This situation creates a need for reliable computer vision-based approaches. While existing soybean seed datasets have been developed for classification tasks, there remains a gap in standardized, high-resolution imagery that captures the subtle morphological differences between Indonesian local varieties under controlled acquisition conditions.

This dataset was compiled to address the specific challenge of distinguishing between visually similar soybean varieties, where morphological differences are often minimal yet agriculturally significant. The motivation behind this work stems from the observation that Indonesian soybean varieties such as Anjasmoro, Grobogan, and DEGA 1 exhibit subtle but measurable differences in seed dimensions and characteristics that are difficult to capture with conventional imaging approaches. By providing a standardized scanner-based dataset with consistent illumination and resolution, this collection enables researchers to develop and benchmark computer vision models specifically tailored for fine-grained cultivar discrimination tasks in agricultural applications.

## 3 Data Description

The dataset contains high-resolution JPG scans of soybean seeds representing three local Indonesian varieties: Anjasmoro, Grobogan, and DEGA 1, accompanied by minimal metadata files. In total, it comprises 119 raw scanned images and 12.683 individual seed instances were extracted through image segmentation. The segmented seeds images are stored in PNG format systematically organized into folders based on their corresponding variety labels.

### 3.1 Directory structure

The dataset is organized into two main components: (1) raw scanned images of soybean seeds and (2) segmented individual seed images.

1. Scanned Images
  The directories Scanned_Anjasmoro/, Scanned_Grobogan/, and Scanned_DEGA_1/ each contain high-resolution JPEG images produced through the scanning process. Within each directory, an images/ subfolder stores the corresponding raw seed scans.
2. Segmented Seeds
  The Segmented_seeds/ directory contains individual seed images obtained through image segmentation and stored in PNG format. Within this directory, segmented seeds are further organized into subfolders by variety, namely segmented_Anjasmoro/, segmented_Grobogan/, and segmented_DEGA_1/.

This hierarchical structure ensures clear separation between raw scanned data and processed segmented images, thereby facilitating ease of access and reproducibility in downstream analyses. The summary of the data is presented in the following Table 1.

**Table 1:** Dataset summary.

Item	Description
Total number of scans	119 JPG images
Per scanned image resolution	Around 6800 × 9359 px
Image size	2.57–2.85 MB
File format	*jpg
Acquisition device	Epson L360 flatbed scanner
Scanner	800 dpi
Calibration	Scanner backlight (ON)
Color space	sRGB
Total number of segmented instances	12.683 soybean seeds across all scans.

### 3.2 File naming convention

Filenames are unique within each folder and follow a simple pattern <variety>_seed_<index>.jpg, for example Anjasmoro_seed_0001.jpg, Grobogan_seed_0002.jpg, Dega_seed_0003.jpg.

### 3.3 Characteristics of varieties

In this study, the primary focus is placed on color based visual features to provide a reference for the expected visual variations in soybean seed images. Although soybean seeds are three dimensional objects, the use of single view images has practical application value because it reflects common machine vision settings used in seed inspection systems, such as flatbed scanners and conveyor-based cameras, which typically operate from a fixed top view. The images are acquired using a realistic and low cost setup that is commonly available in standard laboratories and small scale settings. It ensures that the proposed dataset and acquisition method remain practical, reproducible, and suitable for real world deployment.

The Anjasmoro variety is classified as a large-seeded local soybean with a one-hundred-seed weight ranging from 14.8 to 15.3 *g* The seeds have a yellow seed coat and a yellow-brown hilum. Plants generally grow to a height between 64 and 68 cm and reach maturity within approximately 82 to 92 days. The seeds contain around 41.8 to 42.1 percent protein and 17.1 to 18.6 percent oil [4].

The Grobogan variety is also a large-seeded soybean, with a one-hundred-seed weight of approximately 18 *g* The seeds are light yellow in color with a brown hilum. Plants typically reach a height of 50 to 60 cm, with a maturity period of about 76 days. The average reported yield is 2.77 tons per hectare. Seed composition includes about 43.9 percent protein and 18.4 percent oil. deviation of 3.9 pixels and an average height of 64.3 pixels with a standard deviation of 4.6 pixels.

**Fig. 1:**
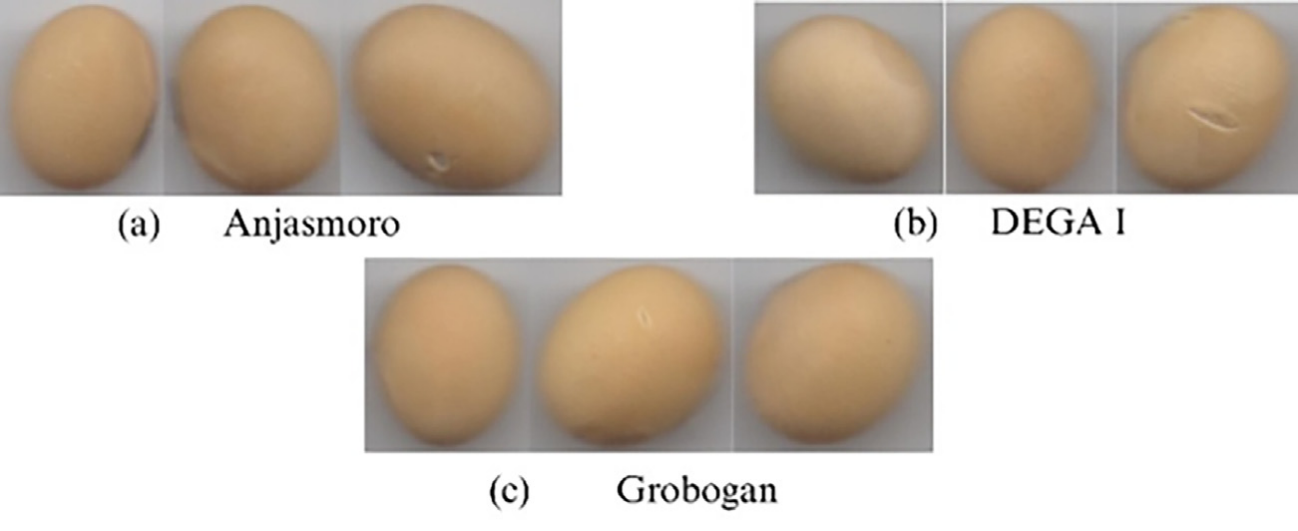
Visual comparison of soybean seed characteristics.

The Dega 1 variety is an early-maturing soybean, typically reaching maturity in 70 to 73 days, with plants growing to a height of about 53 cm. The seeds are large, with a one-hundred-seed weight of 22.98 *g*, and are generally elongated or oval in shape. They have a bright yellowish seed coat and a brown hilum. The average yield is reported to be 2.78 tons per hectare [5]. The differences in the characteristics of the three seed varieties can be observed in Fig. 1.

These varieties traits may manifest in the dataset as size or shape differences at the seed instance level and tone differences around the hilum region, subject to the scanner's illumination and JPEG compression. The datasets used in this research are described in Table 2.

**Table 2:** Dataset description.

Varieties	Seed Instances	Scanned Image	Width Range	Height Range
Anjasmoro	3493	29	180 - 284 pixel	225 - 321 pixel
Dega I	4490	44	179 - 301 pixel	214 - 353 pixel
Grobogan	4700	46	185 - 308 pixel	222 - 360 pixel

## 4 Experimental Design, Materials and Methods

**Fig. 2:**
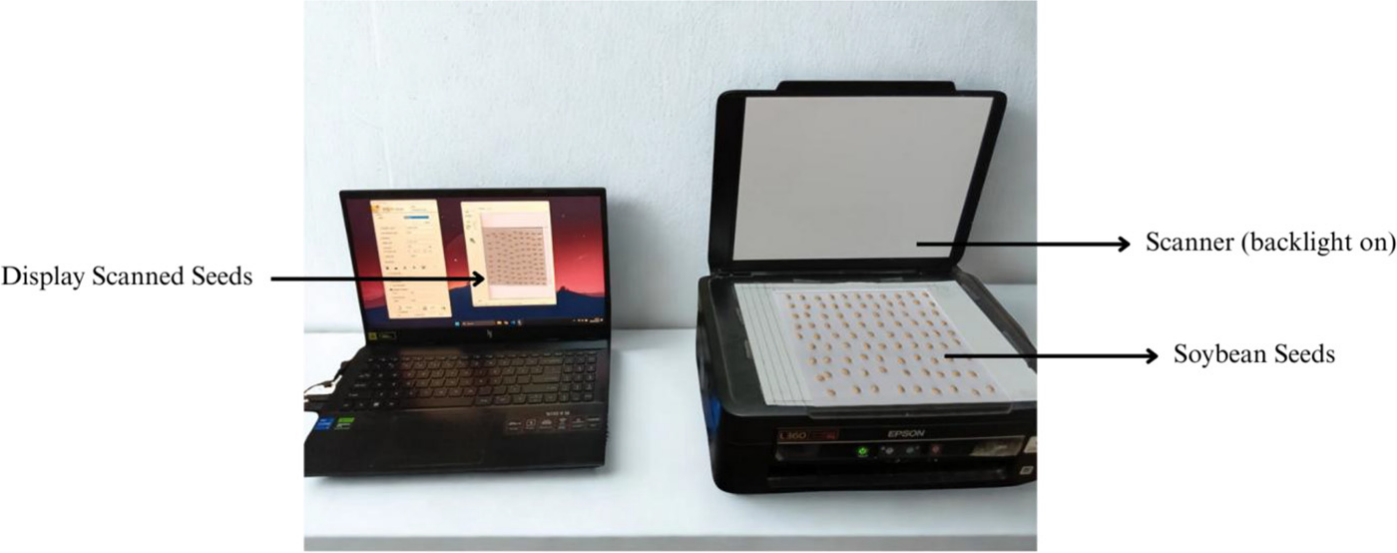
Image acquisition.

The image acquisition process was conducted using an Epson L360 flatbed scanner under standardized conditions. The scanner lamp was consistently enabled during all capture sessions, and the optical resolution was fixed at 800 dots per inch (dpi). Seeds were carefully placed flat on the scanner glass with consistent orientation and positioning to minimize variability. All images were saved directly in JPEG format using the scanner’s default compression settings. This standardized acquisition protocol ensured uniformity across the 119 source scans representing the three soybean varieties, in alignment with established practices for high-throughput seed phenotyping using scanner-based imaging systems [6]. The illustration of data acquisition using the scanner is presented in Fig. 2.

### 4.1 Seed segmentation

**Fig. 3:**
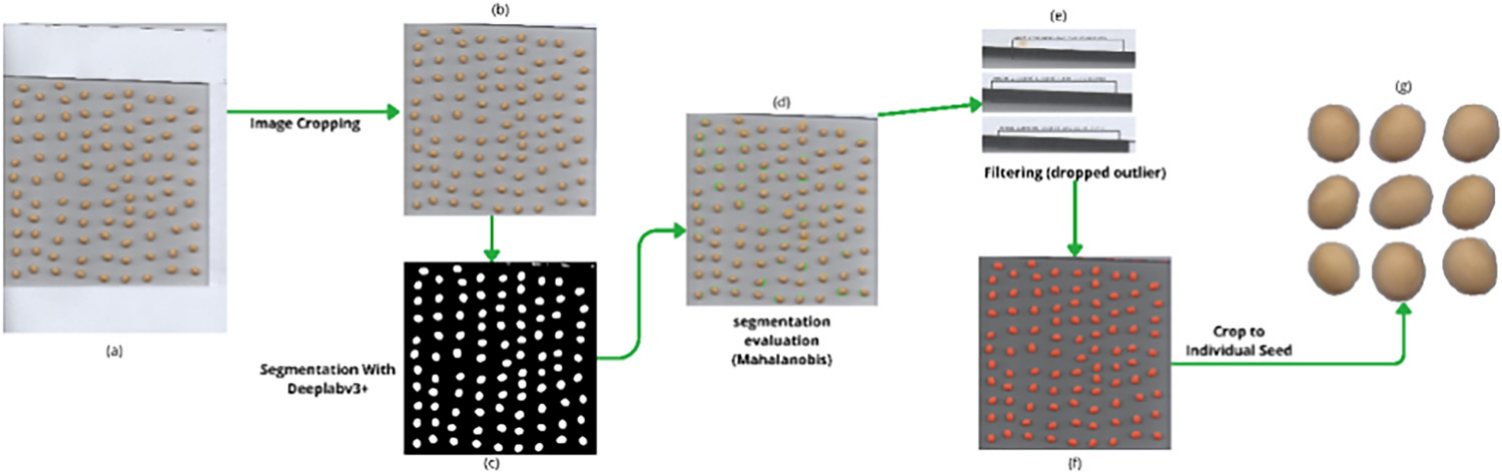
The flowchart of image processing.

The image processing pipeline converted raw scanner outputs into individual seed crops through a multi-stage procedure, following established methodologies in agricultural image analysis for seed phenotyping [7]. Fig. 3 illustrates the workflow to transform from raw scans to the final curated set of cropped seed images. The scanned images, measuring of approximately 6800 × 9359 pixels, first were cropped into a standardized size 6400 × 6400 pixel square region to ensure consistent spatial coverage across samples. Individual seed regions within these cropped images were then identified using a DeepLabv3+ semantic segmentation model with a MobileNet backbone, which has been widely adopted for efficient and high-performance segmentation tasks [8,9]. Image segments that did not meet these criteria were treated as mis-segmentations and will be removed. The remaining valid seed instances were then isolated using mask-aware cropping to generate individual seed images suitable for subsequent analysis.

### 4.2 Evaluation metrics

The segmentation performance was evaluated using mean Intersection over Union (mIoU), which measures the overlap between predicted and ground truth segmentation masks [10]. The Intersection over Union (IoU) is a widely used metric in computer vision that quantifies the accuracy of object detection and segmentation by calculating the ratio of the overlapping area between the predicted segmentation and the ground truth to the total area covered by both regions. IoU values range from 0 to 1, where values closer to 1 indicate better segmentation performance, with a perfect overlap achieving an IoU of 1.0. The mIoU was calculated using Eq. (1).

(1) $$\text{mIoU}=\;\left(\frac{1}{N}\right)\;x\;\sum \left(\text{IoU}\_i\right)$$

N is the number of classes and IoU_i represents the intersection over union for class i. The IoU for each class is computed as the area of intersection divided by the area of union between the predicted and ground truth masks, providing a comprehensive measure of segmentation accuracy across all classes in the dataset.

### 4.3 Experiments method

A comparative evaluation of three backbone architectures was conducted prior for selecting DeepLabv3+. The objective of the selection to optimize the trade-off between segmentation accuracy and computational efficiency, in line with recent advances in lightweight semantic segmentation models for agricultural applications [11]. The performance comparison of the evaluated backbone architectures is summarized in Table 3.

**Table 3:** Performance comparison of different DeepLabv3+ backbone architectures.

Backbone Architecture	Model Size (MB)	Training Epochs	mIoU Score
ResNet-50	155	30	0.9763
ResNet-101	230	35	0.9794
**MobileNet**	**20**	**35**	**0.9701**

Table 3 summarizes the updated performance comparison of the three DeepLabv3+ backbone architectures evaluated in this study. The ResNet-101 backbone achieved the highest segmentation accuracy, with a best validation mIoU of 0.9794 after 35 training epochs, although this improvement came with a substantially larger model size of 230 MB. ResNet-50 reached a slightly lower best mIoU of 0.9763 at 30 epochs with a moderate model size of 155 MB. In contrast, the MobileNet backbone delivered a best mIoU of 0.9701 after 35 epochs while maintaining an exceptionally compact model size of only 20 MB. Although MobileNet exhibits slightly lower accuracy than the residual-network backbones, its drastic reduction in computational and memory overhead offers a favorable trade-off for large-scale phenotyping workflows, aligning with recent trends toward lightweight and resource-efficient architectures in agricultural computer vision [12].

To improve segmentation robustness, a dedicated filtering method was integrated into the preprocessing. This method iteratively eliminated incorrect seed masks using Mahalanobis-distance–based outlier detection in combination with shape-based constraints, such as compared into fill ratio and aspect ratio thresholds. The filtering method was deliberately tuned to favour recall over precision, so that valid seed instances would be preserved despite variability in seed morphology and imaging conditions. Also in seed phenotyping tasks, this emphasis on recall is particularly critical which missing true seed objects can introduce systematic bias into subsequent morphological or colorimetric analyses [13].

**Fig. 4:**
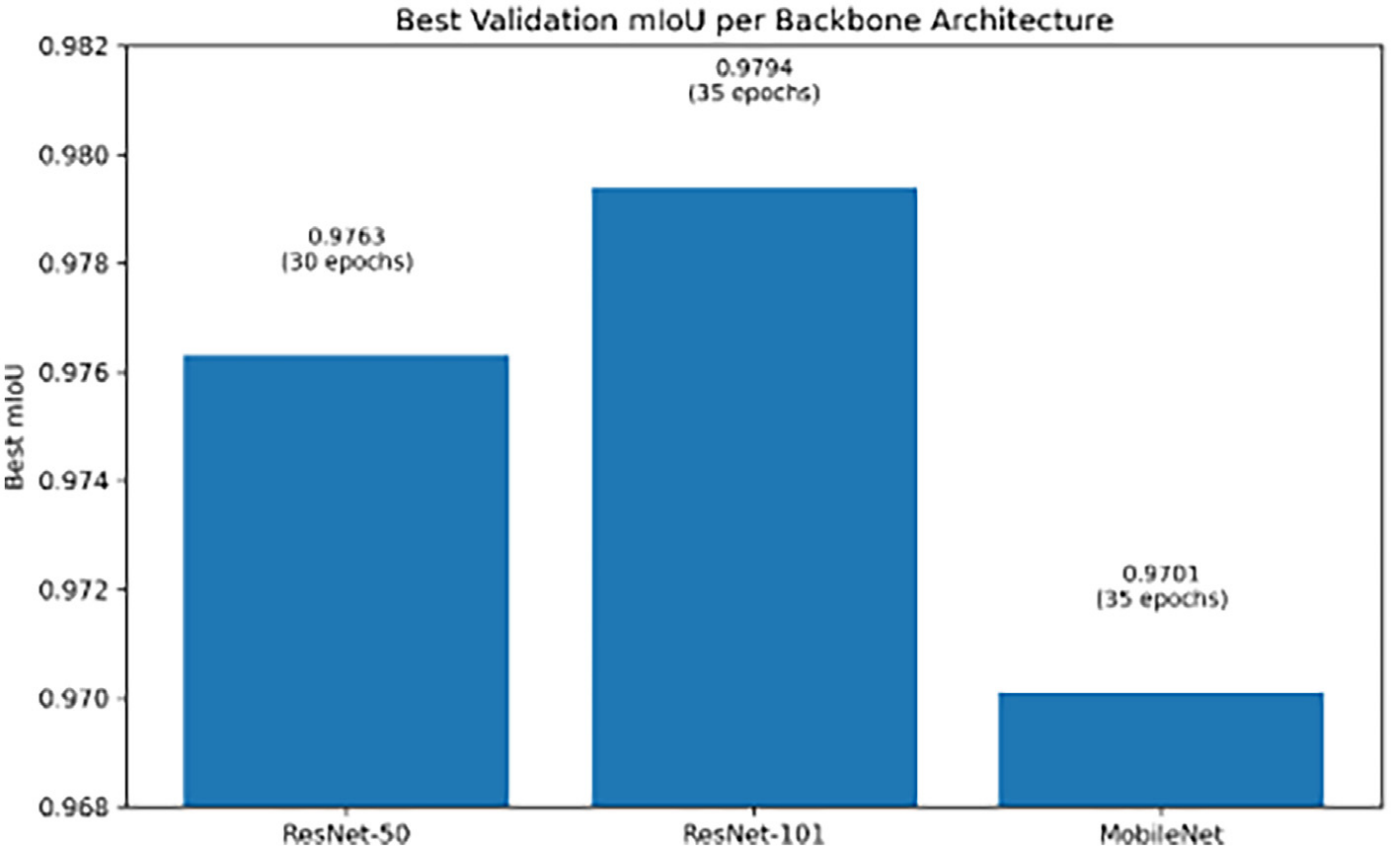
Comparative performance of DeepLabv3+ backbone architectures.

Fig. 4 shows that MobileNet attains a marginally lower mIoU than the residual backbones, but its performance remains consistently stable over training epochs while using a model that is more than seven times smaller. This advantageous trade-off between accuracy and computational efficiency provides strong justification for adopting MobileNet as the backbone of the final segmentation pipeline.

## Limitations

While this dataset provides high-resolution scans and segmented seed images of three widely cultivated local Indonesian soybean varieties (Anjasmoro, Grobogan, and Dega 1), several limitations should be noted. First, the dataset represents only a subset of the broader diversity of Indonesian soybean germplasm, and thus its generalizability to other varieties may be limited. Second, all images were acquired using a single scanner model under controlled laboratory conditions. While this ensures consistency, it does not capture variability that may arise from different imaging devices, environments, or lighting conditions, which could affect the robustness of models trained solely on this dataset. Third, the soybean samples used in the dataset were stored below 16 °C to prevent physical shrinkage and minimize alterations in seed appearance, meaning that dry time and post harvest handling effects were not included as sources of variation. Regarding labeling reliability, all samples were labeled under the supervision of soybean plant breeding experts, which significantly reduces the likelihood of incorrect labels, although minor human errors cannot be entirely ruled out. Furthermore, the dataset does not involve market sourced seeds, as all samples were obtained from controlled cultivation in the Biotechnology Laboratory of the Faculty of Agriculture, Universitas Sumatera Utara. While this eliminates market related biases, it may also limit the diversity of seed conditions commonly found in broader commercial settings. Finally, the morphometric annotations rely solely on automatically extracted features, such as width, height, and aspect ratio, without manual verification, which may introduce minor measurement errors. Despite these considerations, the dataset remains a valuable resource for benchmarking image based seed analysis and provides a standardized foundation for developing and evaluating computer vision models in agricultural research.

## Ethics Statement

The authors have read and follow the ethical requirements for publication in Data in Brief. This work does not involve human participants, animal experiments, or data collected from social media platforms. The dataset consists solely of scanned images of soybean seeds (plant material), and it contains no personally identifiable information or sensitive content

## Credit Author Statement

**Diana Sofia Hanafiah:** Conceptualization, Methodology, Supervision, Resources. **Rahmatika Alfi:** Investigation. **Anggria Lestami:** Project Administration, Resources. **Fanindia Purnamasari:** Methodology, Validation, Writing-Review and Editing. **Rossy Nurhasanah:** Methodology, Validation, Formal Analysis, Writing-Review and Editing. **Muhammad Ariyo Syahraza:** Writing - Original Data Curation, Developing Segmentation Model. **Muhammad Azis Saputra:** Data Preprocessing and Labeling. **Usman Ismail Pane:** Original Data Curation. **Steven Manurung:** Data Preprocessing and Labeling, Software. **Keisya: Model Evaluation. Yunus Tio Buntoro:** Data Curation, **Josua Peter Corda:** Data Curation. **Gali Rakasiwi:** Data Curation.

## Data Availability

Mendeley DataImage Dataset of Local Indonesian Soybean Seed Varieties (Anjasmoro, Grobogan, and DEGA-1) (Original data).

## References

[1] Dolatabadian A., Neik T.X., Danilevicz M.F., Upadhyaya S.R., Batley J., Edwards D. (2025). Image-based crop disease detection using machine learning. Plant Pathol..

[2] Lin W., Fu Y., Xu P., Liu S., Ma D., Jiang Z., Zang S., Yao H., Su Q. (2023). Soybean image dataset for classification. Data Br..

[3] Xu C., Wu T., Yuan S., Sun S., Han T., Song W., Wu C. (2022). Can soybean cultivars with larger seed size produce more protein, lipids, and seed yield? A meta-analysis. Foods.

[4] Fattah A., Idaryani Herniwati, Yasin M., Suriani S., Salim M.B.Nappu, Mulia S., Irawan Hannan M.F., Wulanningtyas H.S., Saenong S., Dewayani W., Suriany E.Winanda, Manwan S.W., Asaad M., Warda Nurjanani, Nurhafsah A.Gaffar, Sunanto A.Y.Fadwiwati, Nurdin M., Dahya A.Ella (2024). Performance and morphology of several soybean varieties and responses to pests and diseases in South Sulawesi. Heliyon.

[5] Kuswantoro H., Ginting E., Yusnawan E., Utomo J.S., Sundari T. (2023). Agronomic performance, seed chemical composition, and bioactive components of selected Indonesian soybean genotypes (*Glycine* max [L.] Merr.). Open Agric..

[6] Tu K., Wu W., Cheng Y., Zhang H., Xu Y., Dong X., Wang M., Sun Q. (2023). AIseed: an automated image analysis software for high-throughput phenotyping and quality non-destructive testing of individual plant seeds. Comput. Electron. Agric..

[7] Huang Z., Wang R., Cao Y., Zheng S., Teng Y., Wang F., Wang L., Du J. (2022). Deep learning based soybean seed classification. Comput. Electron. Agric..

[8] L.-C. Chen, Y. Zhu, G. Papandreou, F. Schroff, H. Adam, Encoder-decoder with atrous separable convolution for semantic image segmentation, in: 2018: pp. 833–851. 10.1007/978-3-030-01234-2_49.

[9] Ghorbani H. (2019). Mahalanobis distance and its application for detecting multivariate outliers. Facta Univ. Ser..

[10] Wang Y., Yang L., Liu X., Yan P. (2024). An improved semantic segmentation algorithm for high-resolution remote sensing images based on DeepLabv3+. Sci. Rep..

[11] Wang Y., Gao X., Sun Y., Liu Y., Wang L., Liu M. (2024). Sh-DeepLabv3+: an improved semantic segmentation lightweight network for corn straw cover form plot classification. Agriculture.

[12] Miao Y., Wang R., Jing Z., Wang K., Tan M., Li F., Zhang W., Han J., Han Y. (2024). CT image segmentation of foxtail millet seeds based on semantic segmentation model VGG16-UNet. Plant Methods.

[13] Xing Y., Lv P., He H., Leng J., Yu H., Feng X. (2022). Traits expansion and storage of soybean phenotypic data in computer vision-based test. Front. Plant Sci..

